# A Novel Sensitive Cell-Based Immunoenzymatic Assay for Palytoxin Quantitation in Mussels

**DOI:** 10.3390/toxins10080329

**Published:** 2018-08-14

**Authors:** Marco Pelin, Silvio Sosa, Valentina Brovedani, Laura Fusco, Mark Poli, Aurelia Tubaro

**Affiliations:** 1 Department of Life Sciences, University of Trieste, 34127 Trieste, Italy; mpelin@units.it (M.P.); ssosa@units.it (S.S.); valebrove@hotmail.it (V.B.); lfusco@units.it (L.F.); 2 U.S. Army Medical Research Institute of Infectious Diseases, Ft. Detrick, MD 21701-5011, USA; mark.a.poli.civ@mail.mil

**Keywords:** Palytoxin, mussels, cell-based ELISA

## Abstract

The marine algal toxin palytoxin (PLTX) and its analogues are some of the most toxic marine compounds. Their accumulation in edible marine organisms and entrance into the food chain represent their main concerns for human health. Indeed, several fatal human poisonings attributed to these compounds have been recorded in tropical and subtropical areas. Due to the increasing occurrence of PLTX in temperate areas such as the Mediterranean Sea, the European Food Safety Authority (EFSA) has suggested a maximum limit of 30 µg PLTX/kg in shellfish meat, and has recommended the development of rapid, specific, and sensitive methods for detection and quantitation of PLTX in seafood. Thus, a novel, sensitive cell-based ELISA was developed and characterized for PLTX quantitation in mussels. The estimated limits of detection (LOD) and quantitation (LOQ) were 1.2 × 10^−11^ M (32.2 pg/mL) and 2.8 × 10^−11^ M (75.0 pg/mL), respectively, with good accuracy (bias = 2.5%) and repeatability (15% and 9% interday and intraday relative standard deviation of repeatability (RSDr), respectively). Minimal interference of 80% aqueous methanol extract allows PLTX quantitation in mussels at concentrations lower than the maximum limit suggested by EFSA, with an LOQ of 9.1 µg PLTX equivalent/kg mussel meat. Given its high sensitivity and specificity, the cell-based ELISA should be considered a suitable method for PLTX quantitation.

## 1. Introduction

Palytoxin (PLTX), a complex marine poly-ol toxin, is one of the most toxic natural compounds. The discovery of PLTX dates back to the 1960s, when, in a tide pool of Hana Bay (Maui Island, Hawaii), Prof. Paul Helfrich collected samples of a toxic soft coral, subsequently identified as *Palythoa toxica*. Ten years later, the chemical structure of PLTX isolated from this coral was reported [1]. Later, PLTX and a series of its analogues were also identified in other Zoantharia belonging to the genera *Palythoa* [2,3,4,5,6,7] and *Zoanthus* [8], in benthic dinoflagellates of the genus *Ostreopsis* [9,10,11,12,13,14,15], and in cyanobacteria of the genus *Trichodesmium* [16]. Only a few of these analogues have been studied from a biological and chemical point of view, including (i) 42-hydroxy-PLTX (42*S*-OH-50*S*-PLTX), isolated from *P. toxica* [2,17], and its stereoisomer (42*S*-OH-50*R*-PLTX), isolated from *P. tuberculosa* [3]; (ii) ostreocin-D (OST-D) and its analogues, produced by *Ostreopsis siamensis* [11,18,19,20]; and (iii) ovatoxin-a (OVTX-a), the most abundant PLTX analogue, produced by *Ostreopsis* cf. *ovata* in the Mediterranean Sea [21,22,23]. 

The main public health concern associated with these toxins is their presence in marine organisms and potential entrance into the human food chain. Indeed, PLTXs have been detected in porifera and polychaete worms as well as in other edible species, including crustaceans, mollusks (gastropods, bivalves, and cephalopods), and echinoderms (sea urchins, starfishes) [8,24,25]. Moreover, consumption of PLTX-contaminated fish or crabs has been associated with cases of fatal human poisoning in tropical and subtropical areas [26,27,28,29]. On the other hand, adverse effects in humans attributed to PLTX along the Mediterranean and Atlantic coasts of Portugal have been associated with inhalation and/or cutaneous exposure to marine aerosol and/or direct exposure to seawater during *Ostreopsis* blooms [30,31]. In particular, signs and symptoms in the respiratory tract, including dyspnea associated with fever >38 °C, as well as conjunctivitis and dermatitis have been reported [13,14,24,27,30,32,33]. In these areas, *Ostreopsis* has been recorded since the early 1970s [34], and in the last decade PLTXs have been detected both in microalgae and in edible marine organisms, but no foodborne poisonings attributed to these toxins have yet been documented. 

Despite their high toxicity, PLTXs are not regulated as seafood or environmental contaminants. However, the European Food Safety Authority (EFSA) has suggested a maximum limit of 30 µg PLTX/kg of shellfish meat [35]. Moreover, given the significant concerns for public health due to the expanding distribution of PLTXs, EFSA has recommended the development of suitable methods to detect these toxins in seafood. In addition to liquid chromatography–mass spectrometry (LC-MS) based chemical methods [36,37], both structural and functional assays are currently available for PLTX. Among these are the hemolytic assay [38,39,40,41,42], the lactate dehydrogenase-based hemolytic biosensor [43], and methods based on PLTX binding to Na^+^/K^+^ ATPase [44,45]. However, these methods suffer from insufficient sensitivity, significant matrix effects, low toxin recovery, and/or other limitations for routine use. Among the structural assays, sensitive, inexpensive, and easy-to-use immunoassays have been set up [46,47,48,49]. Recently, antibody-based biosensors have also been developed as innovative and highly sensitive analytical methods to detect and quantify PLTXs. In particular, a surface plasmon resonance (SPR) biosensor using a murine monoclonal anti-PLTX antibody was set up by Yakes et al. [50], while Zamolo et al. developed a sensitive electrochemiluminescence-based sensor combining the specificity provided by anti-PLTX antibodies and the electric conductivity of carbon nanotubes [51]. Recently, Fraga et al. set up a cytometry immunoassay based on the competitive binding of a monoclonal anti-PLTX antibody between PLTX immobilized on microspheres and PLTX in solution [52]. Another recently developed biosensor for PLTX detection is an immunoenzymatic assay based on biolayer interferometry coupled with a competitive binding assay through an enzyme-linked aptamer [53].

Recently, we demonstrated the ability of specific anti-PLTX antibodies to measure and characterize the binding of PLTX to cultured cells [54]. Using ouabain as a well-known antagonist of PLTX effects in vitro, this binding seems to occur on Na^+^/K^+^ ATPase expressed on the cell surface. Given the high-affinity binding of PLTX to cells and the ability of a monoclonal anti-PLTX antibody to efficiently and simply quantify bound PLTX, a novel cell-based immunoenzymatic assay (cell-based ELISA) for PLTX quantitation was set up and characterized for its sensitivity, accuracy, reproducibility, and specificity. This novel method was further characterized for its suitability to quantify the toxin in mussels.

## 2. Results

### 2.1. Development and Optimization of the Cell-Based ELISA

The cell-based ELISA was developed starting from the protocol for the characterization of PLTX binding to cultured cells reported by Pelin et al. [54]. The assay was then optimized through the following steps: (i) choosing the most sensitive cell line for PLTX binding, (ii) choosing the fixative solutions and temperature of cell incubation with the antibodies, (iii) choosing the sequence of cell fixation and exposure to PLTX, (iv) choosing the blocking agent, and (v) choosing the primary antibody dilution.

#### 2.1.1. PLTX Binding on Different Cultured Cells

**Figure 1:**
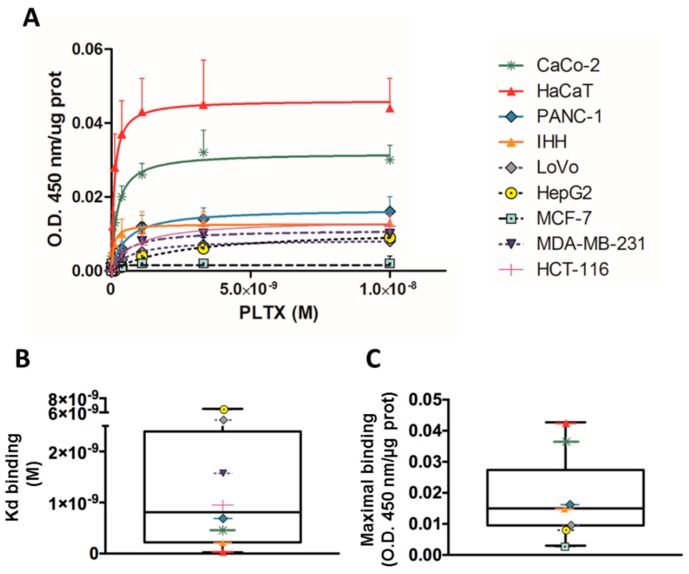
Palytoxin (PLTX) binding evaluated on a panel of different cell lines, detected by a monoclonal mouse anti-PLTX antibody targeted by horseradish peroxidase (HRP)-conjugated anti-mouse immunoglobulin G. (**A**) Saturation curves of PLTX binding. Box plots showing (**B**) distribution of Kd values and (**C**) maximal bindings obtained by the binding assay for PLTX. Results are expressed as mean ± SE of three experiments performed in triplicate.

PLTX binding on cultured cells was evaluated using a panel of different cell lines. Cells were exposed to PLTX for 10 min at 37 °C and the toxin binding was subsequently evaluated as described in the Materials and Methods section. The obtained results for each cell model were normalized on the protein content of each sample. Figure 1 shows the saturation curves of PLTX binding for each cell model (panel A). From these curves, Kd values and maximal binding were calculated, and their distribution was analyzed in the box plot of Figure 1 (panels B and C). A median Kd of 8.1 × 10^−10^ M (interquartile range = 2.2 × 10^−10^ to 2.4 × 10^−9^ M) and a median maximal binding of 0.015 (interquartile range = 0.0095 to 0.02738) were calculated. Binding parameters varied between the different cell lines, and HaCaT cells were the most sensitive cell line, as confirmed by the Kd values and maximal binding (1.4 × 10^−10^ M and 0.043, respectively). On the contrary, the less sensitive cell models for PLTX binding were HepG2 cells (Kd = 6.5 × 10^−9^ M; maximal binding = 0.009) and MCF-7 cells (Kd = not detectable; maximal binding = 0.003). For these reasons, the HaCaT cell line was chosen as the most sensitive model to set up the cell-based ELISA.

#### 2.1.2. Incubation Temperature and Fixing Agents

**Figure 2:**
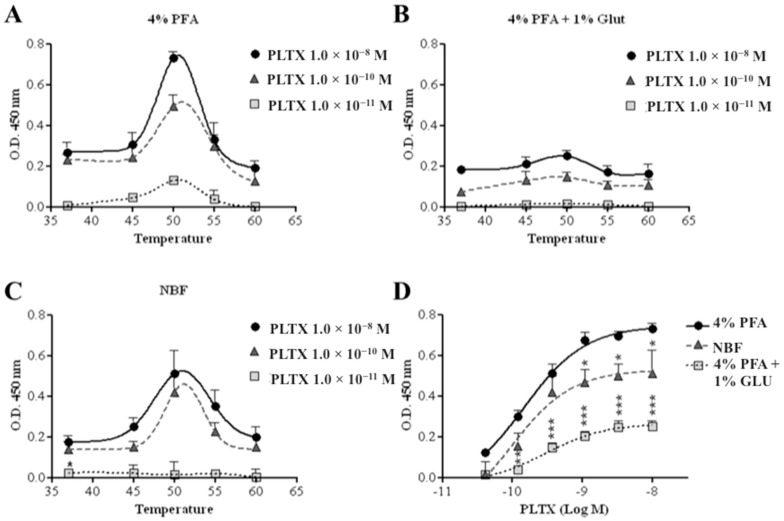
Optimization of the cell-based ELISA. Influence of temperature during cell incubation with the antibodies on the assay signal, using (**A**) 4% paraformaldehyde (PFA), (**B**) 4% PFA + 1% glutaraldehyde, or (**C**) neutral-buffered formalin (NBF) as fixative solutions. (**D**) Influence of the three fixative solutions on the assay signal with antibody incubation at 50 °C. Each point represents mean ± SE of three experiments. Statistical differences: * *p* < 0.05; ** *p* < 0.01; *** *p* < 0.001 (two-way ANOVA and Bonferroni post test).

To improve sensitivity, the assay was carried out exposing HaCaT cells to PLTX, varying the fixing agents and incubation temperature (37–60 °C) with the primary and secondary antibodies. An increased signal (optical density, OD) was observed with increased incubation temperature up to 50 °C, which subsequently decreased at higher temperatures (Figure 2). This trend was recorded also varying the following cell fixing agents: 4% paraformaldehyde (PFA), 4% PFA and 1% glutaraldehyde, neutral-buffered formalin (NBF) (Figure 2).

At the optimal temperature of 50 °C, the highest colorimetric reaction signal was recorded using 4% PFA as fixing agent, followed by NBF (significant differences starting from 1.1 × 10^−9^ M PLTX as compared to the data recorded using 4% PFA, *p* < 0.05) and 4% PFA + 1% glutaraldehyde (significant differences starting from 1.2 × 10^−10^ M PLTX as compared to the data recorded using 4% PFA, *p* < 0.01) (Figure 2D). Thus, 4% PFA and 50 °C were chosen as the optimal fixing agent and incubation temperature with antibodies.

#### 2.1.3. Sequence of Cell Fixation and Exposure to PLTX

**Figure 3:**
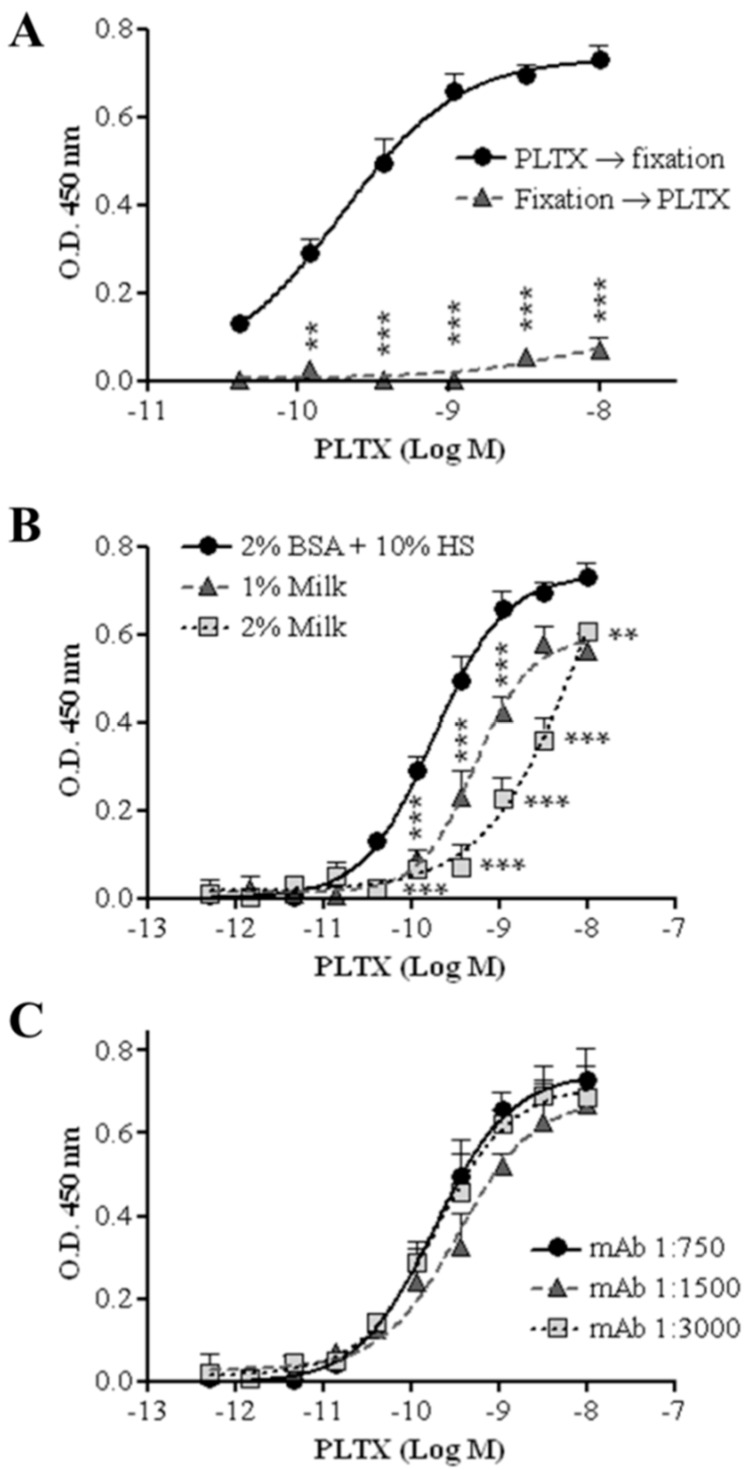
Optimization of the cell-based ELISA. (**A**) Temporal change between the fixation phase (4% PFA) and cell treatment with PLTX (4.1 × 10^−11^–1.0 × 10^−8^ M); (**B**) influence of three blocking agents on the assay signal; (**C**) influence of primary antibody dilution on the assay signal. Each point represents mean ± SE of three experiments. Statistical differences: ** *p* < 0.01; *** *p* < 0.001 (two-way ANOVA and Bonferroni post test).

The possibility of changing the sequence of cell fixation and exposure to PLTX was also evaluated: the cell-based ELISA was carried out fixing HaCaT cells with 4% PFA for 30 min before exposure to PLTX or exposing the cells to the toxin before fixation, as described in the Materials and Methods section. Figure 3A shows the concentration-dependent curve for PLTX detection recorded in the two conditions; the optimal condition consists in cell exposure to PLTX before fixation, since the inverted sequence dramatically decreased the OD values, as expected.

#### 2.1.4. Blocking Agent

The influence of different blocking agents on the assay signal was also evaluated. Figure 3B shows a PLTX calibration curve obtained using three blocking agents. The highest signal was recorded using a Tris-borate buffer (TBB) solution containing 10% horse serum (HS) as a blocking agent. A significant decrease in signal was recorded starting from 1.2 × 10^−10^ M PLTX using a Tris-buffer saline (TBS) solution containing 0.2% Tween 20 and 1% or 2% dried milk powder.

#### 2.1.5. Primary Antibody Dilution

To further increase the signal/background ratio, the influence of different dilutions of the primary antibody on the assay signal was evaluated. As shown in Figure 3C, no significant differences were observed among three primary antibody dilutions tested (1:750, 1:1500, and 1:3000). Thus, the cell-based ELISA was subsequently carried out using the highest dilution (1:3000; 0.5 µg/mL final concentration) of the primary antibody. 

#### 2.1.6. Optimized Cell-Based ELISA

The optimized protocol of the cell-based ELISA consisted of: (i) exposing HaCaT cells (1.5 × 10^4^ cells/well) to PLTX (5.1 × 10^−13^–1.0 × 10^−8^ M) for 10 min at 37 °C, followed by two washes with PBS; (ii) fixation with 4% PFA (50 µL/well) for 30 min, followed by washing with PBS; (iii) blocking with TBB buffer containing 10% HS (200 µL/well) for 30 min, followed by two washes with PBS; (iv) cell incubation with 0.5 µg/mL mouse anti-PLTX monoclonal antibody (mAb) (primary antibody; 100 µL/well) for 1 h at 50 °C under gentle agitation; (v) after three washes with PBS containing 0.1% Tween 20 followed by three washes with PBS, cell incubation with 1:6000 HRP-conjugated anti-mouse IgG (secondary antibody; 100 µL/well) for 1 h at 50 °C under gentle agitation; (vi) after three washes with PBS containing 0.1% Tween 20 and three washes with PBS, cell incubation with tetramethylbenzidine (TMB; 60 µL/well) for 20 min at room temperature; (vii) stopping the colorimetric reaction by 1 M H_2_SO_4_ (30 µL/well); and (viii) measuring the optical density at 450 nm.

### 2.2. Characterization of the Cell-Based ELISA

**Figure 4:**
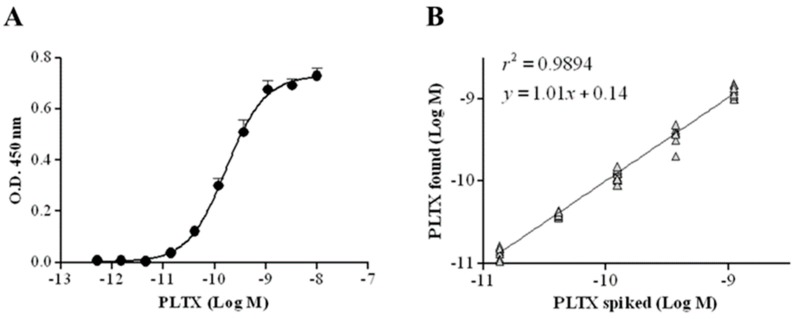
(**A**) Calibration curve of cell-based ELISA for PLTX quantitation. Each point represents mean ± SE of 10 different experiments. (**B**) Linear regression analysis performed within the working range of the cell-based ELISA (1.4 × 10^−11^ to 1.1 × 10^−9^ M) by plotting theoretical PLTX concentrations against toxin concentrations measured by the cell-based ELISA (*n* = 10).

The cell-based ELISA was subsequently characterized by evaluating the limit of detection (LOD) and quantitation (LOQ) of PLTX, as well as the accuracy, repeatability, and specificity of the assay. The calibration curve of PLTX is shown in Figure 4A: the working range is 1.4 × 10^−11^ to 1.1 × 10^−9^ M, whereas the estimated LOD and LOQ of PLTX were 1.2 × 10^−11^ M (32.2 pg/mL) and 2.8 × 10^−11^ M (75.0 pg/mL), respectively. The working range was analyzed by linear regression, plotting the theoretical PLTX concentrations used in the assay against the measured toxin concentrations, and the obtained results revealed a good correlation coefficient (*r*^2^ = 0.9894; *n* = 10) (Figure 4B). A mean bias value of 2.5% (range: −5.1 to 9.8%) was obtained (Table 1).

**Figure 5:**
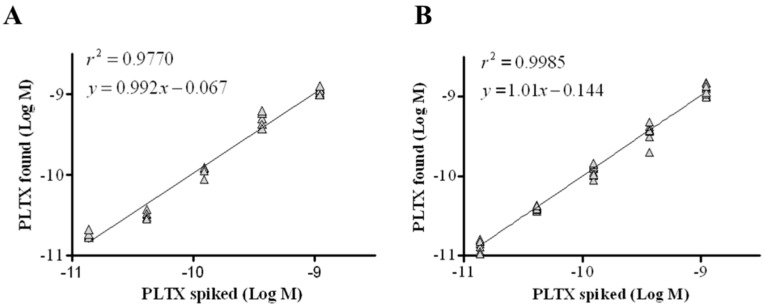
Repeatability of the cell-based ELISA. Linear regression analysis performed within the working range of the assay (1.4 × 10^−11^ to 1.1 × 10^−9^ M) by plotting theoretical PLTX concentrations against toxin concentrations measured by the cell-based ELISA. (**A**) Intraday repeatability (one day, *n* = 6); (**B**) interday repeatability (6 months, *n* = 10).

The intraassay repeatability was estimated over six replicates carried out in one day, while the interassay repeatability was evaluated over 10 replicates carried out over a six-month period. Good correlation coefficients were calculated, with *r*^2^ = 0.9770 for intraassay and *r*^2^ = 0.9985 for interassay (Figure 5). Moreover, the intraday and interday repeatability coefficients (relative standard deviation of repeatability, RSDr) were 12% and 15%, respectively (Table 1).

### 2.3. Cross-Reactivity with Other Marine Toxins

Cross-reactivity was evaluated by analyzing other marine algal toxins structurally unrelated to PLTX that can contaminate seafood: yessotoxin, okadaic acid, domoic acid, brevetoxin-3, saxitoxin, azaspiracid-1, and maitotoxin. These toxins were analyzed by the cell-based ELISA at concentrations ranging from 1 × 10^−12^ M to 1 × 10^−6^ M, and no cross-reactivity was observed (Supplementary Figure S1). 

### 2.4. Inhibition of PLTX Binding by Ouabain

**Figure 6:**
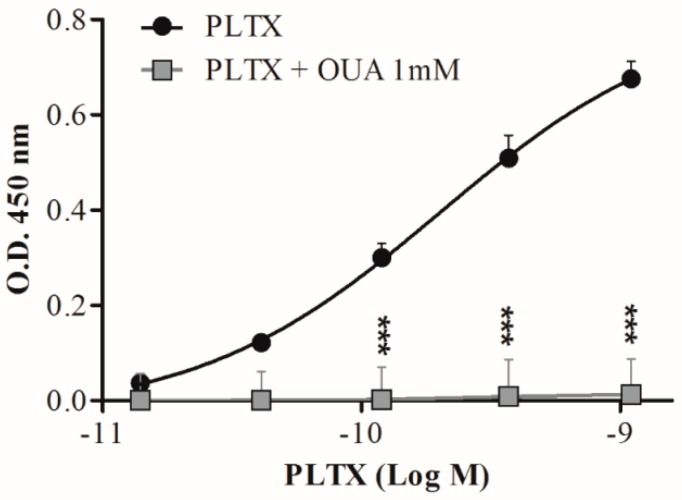
Effect of cell pre-exposure to ouabain on PLTX detection by the cell-based ELISA. Each point represents mean ± SE of three different experiments. Statistical differences: *** *p* < 0.001 as compared to PLTX (two-way ANOVA and Bonferroni post test).

Ouabain (OUA) is a known inhibitor of PLTX in vitro effects due to its binding to the common molecular target, Na^+^/K^+^ ATPase [54,55,56]. Thus, to confirm the specific detection of PLTX by the cell-based ELISA, the assay was performed also exposing the cells to 1 mM OUA for 10 min before their exposure to PLTX (1.4 × 10^−11^ to 1.1 × 10^−9^ M). As can be seen in Figure 6, a significant reduction of the assay signal was recorded when cells were pre-exposed to 1 mM OUA. 

### 2.5. Mussel Matrix Effect

**Figure 7:**
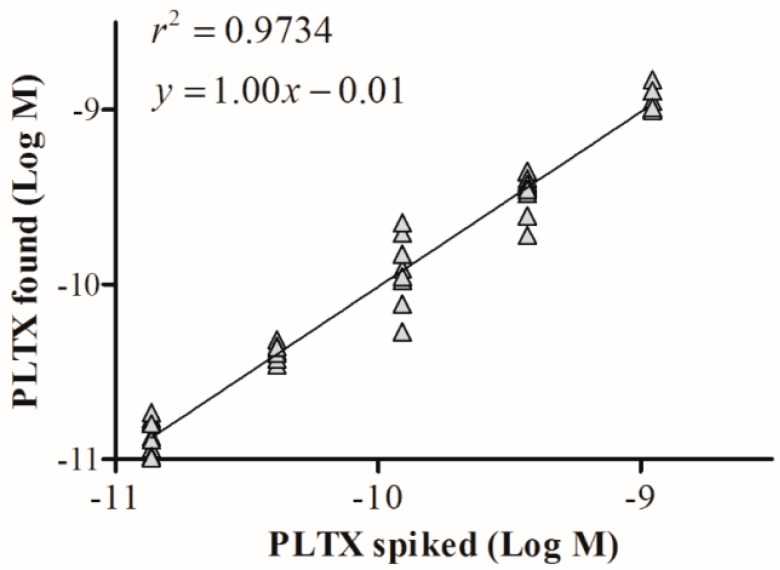
Mussel matrix effect in the cell-based ELISA. Linear regression analysis within the working range of the cell-based ELISA (1.4 × 10^−11^ to 1.1 × 10^−9^ M) performed on 80% aqueous methanol mussel extract diluted 1:10. Linear regression analysis was performed by plotting theoretical PLTX concentrations against toxin concentrations measured by the cell-based ELISA.

The suitability of the cell-based ELISA for quantitation of PLTX in mussels was evaluated by assessing matrix interference on the assay sensitivity. Dilutions (1:2, 1:5, 1:10, 1:50) of the PLTX-free mussel extract in PBS were spiked with known PLTX amounts. Each extract was then analyzed by the cell-based ELISA and the OD values were compared to those obtained by analyzing the same PLTX concentrations without matrix. The minimum extract dilution that did not interfere with the assay was 1:10 (Supplementary Figure S2). At the same dilution, the solvent alone did not interfere with the assay (data not shown). The linear regression analysis between the results obtained by analyzing the 10-fold diluted mussel extract spiked with PLTX and those obtained by analyzing the same PLTX concentrations without matrix (Figure 7) revealed a good correlation coefficient (*r*^2^ = 0.9734) and an excellent mean bias value (mean bias = −1.4%; Table 2). In addition, the estimated LOQ for PLTX in mussels was 3.5 × 10^−11^ M, equal to 9.3 µg/kg mussel meat. 

### 2.6. Recovery of PLTX from Mussels

Recovery experiments were carried out to determine the efficiency of extraction and quantitation of PLTX from mussel meat. Aliquots of PLTX-free mussel homogenate were spiked with PLTX (1.4 × 10^−11^ to 1.1 × 10^−9^ M) and subsequently extracted as described above to obtain extracts containing 0.1 g meat equivalent/mL. Extracts were diluted 1:10 and analyzed by the cell-based ELISA. Recovery of PLTX from mussels ranged between 94% and 110% (coefficient of variability: 13–17%; Table 3).

## 3. Discussion

In recent years, *Ostreopsis* cf. *ovata* has bloomed with increasing frequency in temperate areas such as the Mediterranean Sea and along the Atlantic coast of Portugal [31,34]. Peculiar climate changes and marine conditions, high availability of nutrients, and hydrographic conditions characterized by low wave energy may play important roles in this phenomenon [57]. Concomitant with *Ostreopsis* blooms, PLTXs have been detected in microalgae, aerosolized seawater, and edible marine organisms [30,58,59]. Despite no foodborne poisonings being attributed to PLTX and analogues in this area to date, these toxins can represent a significant public health concern. Therefore, there is a need to develop new detection methods able to quantify PLTXs in seafood at concentrations lower than the maximum limit suggested by EFSA (30 µg PLTX/kg of shellfish meat) [35]. Over the years, various detection methods have been developed, some with limited sensitivity or other limitations, such as significant matrix effects. We developed a cell-based immunoenzymatic assay (cell-based ELISA) that combines the high binding potency of PLTX with the sensitivity and specificity provided by anti-PLTX antibody detection and evaluated it for its ability to accurately quantify PLTX in mussels. 

Initially, the sensitivity of different cell lines to PLTX binding was evaluated to select the most suitable cell line. To this aim, different cell lines derived from colon cancer (LoVo, Caco2, HCT-116), pancreatic cancer (PANC-1), hepatic cancer (HepG2), and breast cancer (MCF-7, MDA-MB-231) were considered. In addition, nontumor cell lines were used, such as the HaCaT skin keratinocyte cell line and the immortalized human hepatic (IHH) cell line. A wide distribution of binding parameters (Kd and maximal binding) was observed, suggesting a wide sensitivity range among the different cell lines. Consequently, the HaCaT cell line turned out to be the most sensitive to PLTX binding, corroborating literature data showing that HaCaT keratinocytes are among the cell models most sensitive to the toxin [60]. For these reasons, this cell line was chosen as the most suitable model to develop the cell-based ELISA. 

To optimize the method, the influence of the incubation temperature of the antibodies on the assay signal as well as the effects of different cell fixing agents were evaluated. The highest signal was obtained using 4% PFA as a fixative solution and incubating the primary and secondary antibodies at 50 °C. The decreased signal recorded after increasing the incubation temperature of the mAb is probably due to a thermal denaturation of the antibodies [61]. For the fixation phase, we chose formaldehyde, a commonly used fixative that easily diffuses into cells, allowing optimal fixation [62]. 

Once the final protocol of the assay was defined, an intralaboratory validation demonstrated good sensitivity, accuracy, and repeatability. As expected, with the combination of two sensitive features, PLTX binding to Na^+^/K^+^ ATPase of HaCaT cells and its detection by monoclonal antibodies, the sensitivity of the cell-based ELISA was higher than that of other PLTX immunoassays [46,48]. In addition, this assay was almost 35 time more sensitive than the indirect sandwich ELISA (estimated LOD = 1.1 ng/mL) developed by our group [47]. With respect to the latter, a significant improvement was achieved in terms of not only sensitivity but also assay time (less than 3 h compared to about 6 h for the sandwich ELISA). Moreover, the sensitivity of the cell-based ELISA, expressed as LOD value (estimated at 1.2 × 10^−11^ M, 32.2 pg/mL) was more than 400 times higher than that of LC-HRMS [36]. Similarly, the estimated LOD was more than 10-fold more sensitive than an SPR-based immunoassay [50] and about twofold more sensitive than the carbon nanotube–based biosensor (70 pg/mL) developed by Zamolo et al. [51]. On the other hand, the sensitivity of the cell-based ELISA was lower than that of the immunoassay (0.5 pg/mL) developed by Garet et al. [49], who employed a single-chain antibody isolated by phage display technology. However, cross-reactivity with other toxins was not investigated and, as discussed later, the method was affected by variable toxin recovery from mussels, hindering its use for PLTX quantitation in this matrix.

The specificity of the cell-based ELISA for PLTX was demonstrated by the lack of cross-reactivity toward other marine algal toxins contaminating seafood [63] and potentially co-occurring with PLTX in edible marine organisms. Moreover, the interference of mussel extract was minimal, as demonstrated by the 1:10 extract dilution that did not interfere with the assay. The 80% aqueous methanol was chosen as an extraction solvent based upon previous studies demonstrating that it is the most suitable for PLTX extraction as assessed by LC-HRMS [64] and the sandwich ELISA [47]. The estimated LOQ of the cell-based ELISA for PLTX in mussels was 9.3 µg PLTX/kg mussel meat, about three times lower than the safety limit suggested by EFSA (30 µg PLTXs/kg of shellfish meat). This result, together with the excellent toxin recovery from mussels (94–110%), suggests the ability of this novel cell-based ELISA to quantify PLTX in mussels with good precision. This is further supported by the good recovery recorded also at very low concentration (1.37 × 10^−11^ M), even lower than that of the estimated LOQ, strengthening the ability of the cell-based ELISA to accurately quantify the toxin in mussels at very low concentrations. 

On the contrary, despite its higher sensitivity, the immunoassay of Garet and colleagues [49] suffers from variable toxin recovery from mussels (64–113%), impairing its suitability for PLTX detection in this matrix. Similarly, significant interference by mussel matrix was observed for other methods, such as the hemolytic assay (LOQ = 640 µg PLTX equivalent/kg mussel meat) [42] and the lactate dehydrogenase–based hemolytic biosensor [43], for which a 1:50 dilution of the mussel extract was necessary. Furthermore, the sensitivity of the cell-based ELISA to quantify PLTX in mussels is higher than that of other antibody-based assays, such as the flow cytometry–based immunoassay (LOQ from 374 to 4430 µg/kg), which required at least a 30-fold extract dilution to avoid matrix effect [50]. In addition, the estimated LOQ of the cell-based ELISA for PLTX in mussels was close to that of other detection methods, such as the sandwich ELISA (11 µg/kg meat) [47], the carbon nanotube–based biosensor (2.2 µg/kg meat) [51], and LC-HRMS (15 µg/kg meat) [36]. However, it must be considered that the cell-based ELISA is a simple, fast, and inexpensive method. Moreover, due to its high sensitivity in buffer solution, it could be further exploited for its suitability to detect and quantify PLTX in other matrices.

## 4. Conclusions

In conclusion, the combination of PLTX binding to Na^+^/K^+^ ATPase with PLTX detection by specific antibodies allowed the development of a novel and sensitive cell-based immunoenzymatic assay for PLTX detection. This combination resulted in higher sensitivity as compared to other very sensitive methods. Hence, the cell-based ELISA described here is sensitive, repeatable, and accurate, and is able to quantify PLTX in mussels at concentrations lower than the maximum limit suggested by EFSA (30 µg PLTX/kg shellfish meat). Thus, the high sensitivity, specificity, and rapidity make the cell-based ELISA a suitable method for PLTX screening in mussels during monitoring programs. Further studies are in progress to characterize the ability of the cell-based ELISA to detect and quantify PLTX in other frequently contaminated matrices, such as other shellfish, fish, crabs, and other seafood.

## 5. Materials and Methods 

### 5.1. Chemicals

Palytoxin, isolated from *Palythoa tuberculosa* (purity > 90%), was purchased from Wako Pure Chemicals Industries Ltd. (Osaka, Japan). Yessotoxin and maitotoxin were kindly provided by Prof. T. Yasumoto (Japan Food Research Laboratories, Tokyo, Japan). Brevetoxin-3, saxitoxin, and azaspiracid-1 were supplied by Dr. M. Poli (U.S. Army Medical Research Institute of Infectious Diseases, Ft. Detrick, MD, USA), Dr. F. Van Dolah (National Oceanic and Atmospheric Administration, Charleston, SC, USA), and Dr. J. Kilcoyne (Marine Institute, Rinville, Oranmore, County Galway, Ireland), respectively. Okadaic and domoic acids were purchased from Sigma-Aldrich (Milan, Italy). The mouse monoclonal anti-palytoxin antibody 73D3 (mAb-PLTX) was produced and purified from a hybridoma cell culture at the U.S. Army Medical Research Institute of Infectious Diseases (Ft. Detrick, MD, USA). The horseradish peroxidase (HRP)-conjugated anti-mouse immunoglobulin G (IgG) was purchased from Jackson ImmunoResearch (Newmarket, UK). HaCaT cell line was purchased from Cell Line Service (DKFZ, Eppelheim, Germany), and all cell culture reagents were purchased from EuroClone (Milan, Italy). All other reagents were of analytical grade and purchased from Sigma-Aldrich (Milan, Italy).

### 5.2. Cell Cultures

HaCaT, PANC-1, and HCT-116 cells were cultured in Dulbecco’s Modified Eagle’s Medium (DMEM) high glucose, supplemented with 10% fetal bovine serum, L-glutamine (1.0 × 10^−2^ M), penicillin (1.0 × 10^−4^ g/mL), and streptomycin (1.0 × 10^−4^ g/mL). HepG2 and Caco2 cells were maintained in Eagle’s Minimal Essential Medium (EMEM) with the addition of 10% fetal bovine serum (FBS), L-glutamine (1.0 × 10^−2^ M), penicillin (1.0 × 10^−4^ g/mL) and streptomycin (1.0 × 10^−4^ g/mL), and 1% sodium pyruvate 100 mM. LoVo, MCF-7, and MDA-MB-231 cells were maintained in RPMI 1640 containing 10% FBS, L-glutamine (1.0 × 10^−2^ M), penicillin (1.0 × 10^−4^ g/mL), and streptomycin (1.0 × 10^−4^ g/mL). IHH cells were maintained in DMEM medium-high glucose with the addition of 10% FBS, 1.25% L-glutamine 200 mM, penicillin (1.0 × 10^−4^ g/mL) and streptomycin (1.0 × 10^−4^ g/mL), 1% HEPES buffer 1 M, 0.01% human insulin 10^−4^ M, and 0.04% dexamethasone 1 mg/mL.

Cell cultures were maintained according to standard procedures in a humidified incubator at 37 °C with 5% CO_2_, and cell passage was performed at confluence once per week.

### 5.3. Experimental Design

- *Development of the cell-based ELISA.* The cell-based ELISA was developed starting from the protocol used to characterize PLTX binding to cultured cells reported by Pelin et al. [54]. Cells were seeded in 96-well plates (1.5 × 10^4^ cells/well) and cultured for 3 days. The assay procedure consisted of the following steps: (i) cell exposure to PLTX (5.1 × 10^−13^ to 1.0 × 10^−8^ M) for 10 min at 37 °C, followed by washing away unbound toxin with PBS; (ii) cell fixation with 4% PFA for 30 min; (iii) blocking with TBB buffer (50 mM Tris-HCl, 0.15 M NaCl, 2% bovine serum albumin (BSA), and 0.2% Tween 20, pH 7.5) containing 10% HS for 30 min, followed by washing with PBS; (iv) incubation with mouse monoclonal anti-PLTX antibody (2 µg/mL; primary antibody) for 1 h at room temperature (RT) followed by washing with PBS containing 0.1% Tween 20 and PBS; (v) incubation with 1:6000 HRP-conjugated anti-mouse IgG (secondary antibody) for 1 h at 37 °C, followed by washing with PBS containing 0.1% Tween 20 and PBS; (vi) incubation with 60 µL/well of tetramethylbenzidine (TMB) chromogen and hydrogen peroxide substrate for 20 min; (vii) stopping the colorimetric reaction by 1 M H_2_SO_4_ (30 µL/well); and (viii) measuring the optical density at 450 nm (Spectra^®^ photometer, Tecan Italia, Milan, Italy).
- *Assay optimization.* The assay was optimized by varying the following parameters: (i) type of cell line, (ii) fixative agents and temperature of incubation with primary and secondary antibodies, (iii) sequence of cell fixation and cell exposure to PLTX, (iv) blocking agents, and (v) dilution of the primary antibody.
- *Assay characterization.* The optimized assay was characterized following the Eurachem Guide [64] as described in the Statistical Analysis section.

### 5.4. Evaluation of Mussel Matrix Effect

The suitability of the cell-based ELISA for PLTX quantitation in mussels was assessed using different extracts from edible parts of *Mytilus galloprovincialis* collected in the Gulf of Trieste (Trieste, Italy). Shucked mussel meat (200 g) was homogenized using an Ultra-Turrax (Ika-Werk; Staufen, Germany) at 14,000 rpm and room temperature, until a homogeneous pulp was obtained (about 5 min). Mussel homogenate (1 g) was extracted 3 times with 3 mL 80% aqueous MeOH by Ultra-Turrax homogenization (14,000 rpm, 3 min) followed by centrifugation at 5500 rpm for 30 min. The supernatants were then pooled and the volume adjusted to 10 mL with 80% aqueous methanol to obtain 0.1 g mussel meat equivalent/mL. The extract was analyzed by LC-MS/MS (Thermo-Fisher, San Josè, CA, USA) to confirm the absence of PLTX before the matrix effect evaluation. Then, dilutions (1:2, 1:5, 1:10, and 1:50 in PBS, *v/v*) of the PLTX-free mussel extract were spiked with known PLTX concentrations to prepare a series of matrix matched samples at PLTX concentrations ranging from 1.4 × 10^−11^ M to 1.1 × 10^−9^ M. These samples were then analyzed by the cell-based ELISA and compared to the same PLTX concentrations in solutions free of mussel matrix.

### 5.5. Evaluation of PLTX Recovery from Mussels

To evaluate the recovery of PLTX from mussels, samples of the PLTX-free mussel homogenate were spiked with known amounts of PLTX and then extracted as described above to obtain extracts containing different theoretic concentrations of PLTX (1.4 × 10^−11^ to 1.1 × 10^−9^ M). Each extract was then analyzed by the cell-based ELISA, as previously described.

### 5.6. Statistical Analysis

PLTX concentrations are reported as mean ± SE of at least 3 independent experiments performed in triplicate, unless otherwise specified. For binding experiments, dissociation constant (Kd) was calculated by a 1-site binding hyperbola nonlinear regression analysis using GraphPad Prism software version 6.0 (GraphPad, Inc., San Diego, CA, USA). Maximal binding was evaluated as the maximal optical density (OD) normalized for the µg of proteins of each sample. Linearity (*r*^2^) of the calibration curve was estimated by linear regression analysis, also using GraphPad Prism version 6.0. Data were compared by 2-way ANOVA followed by Bonferroni post test, and significant differences were considered at *p* values < 0.05.

The optimized assay was characterized according to the international principles defined by the Eurachem Guide [65]: limits of detection (LOD) and quantitation (LOQ) were estimated as PLTX concentration corresponding to the average of the optical density of 10 blank values plus 3 or 10 times the standard deviation, respectively. Repeatability was expressed as relative standard deviation of repeatability (RSDr), measured as % ratio between the standard deviation of independent results and their mean value. Independent results obtained by the same operator in 1 day (intraassay RSDr; *n* = 6) and by different operators within a 6-month period (interassay RSDr; *n* = 10) were evaluated. Accuracy was measured as % bias (*n* = 10), calculated as % difference between PLTX concentration measured by the assay and the theoretical concentration in the sample divided for theoretical PLTX concentrations.

## Figures and Tables

**Table 1:** Bias values (%) for PLTX analysis by the cell-based ELISA, intraday (*n* = 6, 1 day) and interday (*n* = 10, 6 months) repeatability (relative standard deviation of repeatability, RSDr %), and mean of PLTX concentrations measured by the assay.

Theoretical PLTX Concentration (M)	Bias (%)	Intraday Repeatability		Interday Repeatability	
		Mean of Measured PLTX Concentration (M)	RSDr (%)	Mean (M)	RSDr (%)
1.37 × 10^−11^	6.9	1.83 × 10^−11^	8	1.42 × 10^−11^	14
4.12 × 10^−11^	−5.1	3.22 × 10^−11^	10	3.94 × 10^−11^	6
1.24 × 10^−10^	−1.6	1.15 × 10^−10^	12	1.21 × 10^−10^	15
3.70 × 10^−10^	2.6	5.01 × 10^−10^	21	3.80 × 10^−10^	21
1.11 × 10^−9^	9.8	1.11 × 10^−9^	10	1.22 × 10^−9^	16
Mean	2.5		12		15

**Table 2:** Bias values (%) for PLTX detected in 80% aqueous methanol mussel extract spiked with the toxin after 1:10 dilution compared to theoretical PLTX concentrations (*n* = 10).

PLTX Concentration (M)	Bias (%)
1.37 × 10^−11^	−0.3
4.12 × 10^−11^	−0.6
1.23 × 10^−10^	1.5
3.70 × 10^−10^	−4.8
1.11 × 10^−9^	−3.0
Mean	−1.4

**Table 3:** Recovery of PLTX from mussels analyzed by the cell-based ELISA.

PLTX Concentration (M)	Recovery (%)	Coefficient of Variability (%)	No. of Replicas
1.37 × 10^−11^	99.6	15	5
4.12 × 10^−11^	101.0	13	5
1.23 × 10^−10^	93.7	17	5
3.70 × 10^−10^	110.2	14	5
1.11 × 10^−9^	101.9	13	5

## Supplementary Materials

The following are available online at http://www.mdpi.com/2072-6651/10/8/329/s1. Figure S1: Cross-reactivity of cell-based ELISA with other marine toxins, Figure S2: Evaluation of mussel matrix effect on cell-based ELISA.

## References

[1] Moore R.E., Scheuer P.J. (1971). Palytoxin: A new marine toxin from a coelenterate. Science.

[2] Ciminiello P., Dell’Aversano C., Dello Iacovo E., Fattorusso E., Forino M., Grauso L., Tartaglione L., Florio C., Lorenzon P., De Bortoli M. (2009). Stereostructure and biological activity of 42-Hydroxy-palytoxin: A new palytoxin analogue from Hawaiian *Palythoa* subspecies. Chem. Res. Toxicol..

[3] Ciminiello P., Dell’Aversano C., Dello Iacovo E., Forino M., Tartaglione L., Pelin M., Sosa S., Tubaro A., Chaloin O., Poli M. (2014). Stereoisomers of 42-hydroxy palytoxin from Hawaiian *Palythoa toxica* and *P. tuberculosa*: Stereostructure elucidation, detection, and biological activities. J. Nat. Prod..

[4] Deeds J.R., Handy S.M., White K.D., Reimer J.D. (2011). Palytoxin found in *Palythoa* sp. zoanthids (Anthozoa, Hexacorallia) sold in the home aquarium trade. PLoS ONE.

[5] Tartaglione L., Pelin M., Morpurgo M., Dell’Aversano C., Montenegro J., Sacco G., Sosa S., Reimer J.D., Ciminiello P., Tubaro A. (2016). An aquarium hobbyist poisoning: Identification of new palytoxins in *Palythoa* cf. toxica and complete detoxification of the aquarium water by activated carbon. Toxicon.

[6] Pelin M., Brovedani V., Sosa S., Tubaro A. (2016). Palytoxin-Containing Aquarium Soft Corals as an Emerging Sanitary Problem. Mar. Drugs.

[7] Fraga M., Vilariño N., Louzao M.C., Molina L., López Y., Poli M., Botana L.M. (2017). First Identification of Palytoxin-Like Molecules in the Atlantic Coral Species Palythoa canariensis. Anal. Chem..

[8] Gleibs S., Mebs D., Werding B. (1995). Studies on the origin and distribution of palytoxin in a Caribbean coral reef. Toxicon.

[9] Ciminiello P., Dell’Aversano C., Fattorusso E., Forino M., Tartaglione L., Grillo C., Melchiorre N. (2008). Putative palytoxin and its new analogue, ovatoxin-a, in *Ostreopsis ovata* collected along the Ligurian coasts during the 2006 toxic outbreak. J. Am. Soc. Mass. Spectrom.

[10] Lenoir S., Ten-Hage L., Turquet J., Quod J.P., Bernard C., Hennion M.C. (2004). First evidence of palytoxin analogues from an *Ostreopsis mascarenensis* (Dinophyceae) bentic bloom in Southwestern Indian Ocean. J. Phycol..

[11] Ukena T., Satake M., Usami M., Oshima Y., Naoki H., Fujita T., Kan Y., Yasumoto T. (2001). Structure elucidation of ostreocin D, a palytoxin analog isolated from the dinoflagellate *Ostreopsis siamensis*. Biosci. Biotechnol. Biochem..

[12] Garcia-Altares M., Tartaglione L., Dell’Aversano C., Carnicer O., De la Iglesia P., Forino M., Diogene J., Ciminiello P. (2015). The novel ovatoxin-g and isobaric palytoxin (so far referred to as putative palytoxin) from *Ostreopsis* cf. *ovata* (NW Mediterranean Sea): Structural insights by LC-high resolution MS. Anal. Bioanal. Chem..

[13] Brissard C., Herrenknecht C., Sechet V., Herve F., Pisapia F., Harcouet J., Lemee R., Chomerat N., Hess P., Amzil Z. (2014). Complex toxin profile of French Mediterranean *Ostreopsis cf. ovata* strains, seafood accumulation and ovatoxins prepurification. Mar. Drugs.

[14] Amzil Z., Sibat M., Chomerat N., Grossel H., Marco-Miralles F., Lemee R., Nezan E., Sechet V. (2012). Ovatoxin-a and palytoxin accumulation in seafood in relation to *Ostreopsis cf. ovata* blooms on the French Mediterranean coast. Mar. Drugs.

[15] Rossi R., Castellano V., Scalco E., Serpe L., Zingone A., Soprano V. (2010). New palytoxin-like molecules in Mediterranean *Ostreopsis cf. ovata* (dinoflagellates) and in *Palythoa tuberculosa* detected by liquid chromatography-electrospray ionization time-of-flight mass spectrometry. Toxicon.

[16] Kerbrat A.S., Amzil Z., Pawlowiez R., Golubic S., Sibat M., Darius H.T., Chinain M., Laurent D. (2011). First evidence of palytoxin and 42-hydroxy-palytoxin in the marine cyanobacterium *Trichodesmium*. Mar. Drugs.

[17] Tubaro A., Del Favero G., Beltramo D., Ardizzone M., Forino M., De Bortoli M., Pelin M., Poli M., Bignami G., Ciminiello P. (2011). Acute oral toxicity in mice of a new palytoxin analog: 42-hydroxy-palytoxin. Toxicon.

[18] Ito E., Yasumoto T. (2009). Toxicological studies on palytoxin and ostreocin-D administered to mice by three different routes. Toxicon.

[19] Ares I.R., Cagide E., Louzao M.C., Espiña B., Vieytes M.R., Yasumoto T., Botana L.M. (2009). Ostreocin-D impact on globular actin of intact cells. Chem. Res. Toxicol..

[20] Usami M., Satake M., Ishida S., Inoue A., Kan Y., Yasumoto T. (1995). Palytoxin analogs from the dinoflagellate *Ostreopsis siamensis*. J. Am. Chem. Soc..

[21] Ciminiello P., Dell’Aversano C., Dello Iacovo E., Fattorusso E., Forino M., Grauso L., Tartaglione L., Guerrini F., Pezzolesi L., Pistocchi R. (2012). Isolation and structure elucidation of ovatoxin-a, the major toxin produced by *Ostreopsis ovata*. J. Am. Chem. Soc..

[22] Pelin M., Forino M., Brovedani V., Tartaglione L., Dell’Aversano C., Pistocchi R., Poli M., Sosa S., Florio C., Ciminiello P. (2016). Ovatoxin-a, A palytoxin analogue isolated from *Ostreopsis cf. ovata* Fukuyo: Cytotoxic activity and ELISA detection. Environ. Sci. Technol..

[23] Poli M., Ruiz-Olvera P., Nalca A., Ruiz S., Livingston V., Frick O., Dyer D., Schellhase C., Raymond J., Kulis D. (2018). Toxicity and pathophysiology of palytoxin congeners after intraperitoneal and aerosol administration in rats. Toxicon.

[24] Biré R., Trotereau S., Lemée R., Delpont C., Chabot B., Aumond Y., Krys S. (2013). Occurrence of palytoxins in marine organisms from different trophic levels of the French Mediterranean coast harvested in 2009. Harmful Algae.

[25] Gleibs S., Mebs D. (1999). Distribution and sequestration of palytoxin in coral reef animals. Toxicon.

[26] Deeds J.R., Schwartz M.D. (2010). Human risk associated with palytoxin exposure. Toxicon.

[27] Tubaro A., Durando P., Del Favero G., Ansaldi F., Icardi G., Deeds J.R., Sosa S. (2011). Case definitions for human poisonings postulated to palytoxins exposure. Toxicon.

[28] Wu M.L., Yang C.C., Deng J.F., Wang K.Y. (2014). Hyperkalemia, hyperphosphatemia, acute kidney injury, and fatal dysrhythmias after consumption of palytoxin contaminated goldspot herring. Ann. Emerg. Med..

[29] Patocka J., Nepovimova E., Wu Q., Kuca K. (2018). Palytoxin congeners. Arch. Toxicol..

[30] Del Favero G., Sosa S., Pelin M., D’Orlando E., Florio C., Lorenzon P., Poli M., Tubaro A. (2012). Sanitary problems related to the presence of *Ostreopsis* spp. in the Mediterranean Sea: A multidisciplinary scientific approach. Ann. Ist. Super. Sanita.

[31] Silva M., Pratheepa V.K., Botana L.M., Vasconcelos V. (2015). Emergent toxins in North Atlantic temperate waters: A challenge for monitoring programs and legislation. Toxins (Basel).

[32] Durando P., Ansaldi F., Oreste P., Moscatelli P., Marensi L., Grillo C., Gasparini R., Icardi G. (2007). Collaborative Group for the Ligurian Syndromic Algal Surveillance. *Ostreopsis ovata* and human health: Epidemiological and clinical features of respiratory syndrome outbreaks from a two-year syndromic surveillance, 2005-06, in north-west Italy. Euro Surveill..

[33] Tichadou L., Glaizal M., Armengaud A., Grossel H., Lemée R., Kantin R., Lasalle J.L., Drouet G., Rambaud L., Malfait P. (2010). Health impact of unicellular algae of the *Ostreopsis* genus blooms in the Mediterranean Sea: Experience of the French Mediterranean coast surveillance network from 2006 to 2009. Clin. Toxicol..

[34] Rhodes L. (2011). World-wide occurrence of the toxic dinoflagellate genus *Ostreopsis* Schmidt. Toxicon.

[35] EFSA (2009). Scientific Opinion on marine biotoxins in shellfish-Palytoxin group: Marine Biotoxins in Shellfish-Palytoxin group. EFSA J..

[36] Ciminiello P., Dell’Aversano C., Dello Iacovo E., Forino M., Tartaglione L. (2015). Liquid chromatography-high-resolution mass spectrometry for palytoxins in mussels. Anal. Bioanal. Chem..

[37] Wunschel D.S., Valenzuela B.R., Kaiser B.L.D., Victry K., Woodruff D. (2018). Method development for comprehensive extraction and analysis of marine toxins: Liquid-liquid extraction and tandem liquid chromatography separations coupled to electrospray tandem mass spectrometry. Talanta.

[38] Habermann E., Ahnert-Hilger G., Chhatwal G.S., Beress L. (1981). Delayed haemolytic action of palytoxin. General characteristics. Biochim. Biophys. Acta.

[39] Bignami G.S. (1993). A rapid and sensitive hemolysis neutralization assay for palytoxin. Toxicon.

[40] Riobò P., Paz B., Franco J.M., Vazquez J.A., Murado M.A. (2008). Proposal for a simple and sensitive haemolytic assay for palytoxin: Toxicological dynamics, kinetics, ouabain inhibition and thermal stability. Harmful Algae.

[41] Seemann P., Gernert C., Schmitt S., Mebs D., Hentschel U. (2009). Detection of hemolytic bacteria from *Palythoa caribaeorum* (Cnidaria, Zoantharia) using a novel palytoxin-screening assay. Antonie Van Leeuwenhoek.

[42] Brovedani V., Sosa S., Poli M., Forino M., Varello K., Tubaro A., Pelin M. (2016). A revisited hemolytic assay for palytoxin detection: Limitations for its quantitation in mussels. Toxicon.

[43] Volpe G., Cozzi L., Migliorelli D., Croci L., Palleschi G. (2014). Development of a haemolytic-enzymatic assay with mediated amperometric detection for palytoxin analysis: Application to mussels. Anal. Bioanal. Chem..

[44] Alfonso A., Fernández-Araujo A., Alfonso C., Caramés B., Tobio A., Louzao M.C., Vieytes M.R., Botana L.M. (2012). Palytoxin detection and quantification using the fluorescence polarization technique. Anal. Biochem..

[45] Alfonso A., Pazos M.J., Fernández-Araujo A., Tobio A., Alfonso C., Vieytes M.R., Botana L.M. (2013). Surface plasmon resonance biosensor method for palytoxin detection based on Na^+^,K^+^-ATPase affinity. Toxins.

[46] Bignami G.S., Raybould T.J., Sachinvala N.D., Grothaus P.G., Simpson S.B., Lazo C.B., Byrnes J.B., Moore R.E., Vann D.C. (1992). Monoclonal antibody-based enzyme-linked immunoassays for the measurement of palytoxin in biological samples. Toxicon.

[47] Boscolo S., Pelin M., De Bortoli M., Fontanive G., Barreras A., Berti F., Sosa S., Chaloin O., Bianco A., Yasumoto T. (2013). Sandwich ELISA assay for the quantitation of palytoxin and its analogs in natural samples. Environ. Sci. Technol..

[48] Frolova G.M., Kuznetsova T.A., Mikhaĭlov V.V., Eliakov G.B. (2000). Immunoenzyme method for detecting microbial producers of palytoxin. Bioorg. Khim..

[49] Garet E., Cabado A.G., Vieites J.M., González-Fernández A. (2010). Rapid isolation of single-chain antibodies by phage display technology directed against one of the most potent marine toxins: Palytoxin. Toxicon.

[50] Yakes B.J., DeGrasse S.L., Poli M., Deeds J.R. (2011). Antibody characterization and immunoassays for palytoxin using an SPR biosensor. Anal. Bioanal. Chem..

[51] Zamolo V.A., Valenti G., Venturelli E., Chaloin O., Marcaccio M., Boscolo S., Castagnola V., Sosa S., Berti F., Fontanive G. (2012). Highly sensitive electrochemiluminescent nanobiosensor for the detection of palytoxin. ACS Nano.

[52] Fraga M., Vilariño N., Louzao M.C., Fernández D.A., Poli M., Botana L.M. (2016). Detection of palytoxin-like compounds by a flow cytometry-based immunoassay supported by functional and analytical methods. Anal. Chim. Acta.

[53] Gao S., Zheng X., Hu B., Sun M., Wu J., Jiao B., Wang L. (2017). Enzyme-linked, aptamer-based, competitive biolayer interferometry biosensor for palytoxin. Biosens. Bioelectron..

[54] Pelin M., Boscolo S., Poli M., Sosa S., Tubaro A., Florio C. (2013). Characterization of palytoxin binding to HaCaT cells using a monoclonal anti-palytoxin antibody. Mar. Drugs.

[55] Habermann E., Chhatwal G.S. (1982). Ouabain inhibits the increase due to palytoxin of cation permeability of erythrocytes. Naunyn Schmiedebergs Arch. Pharmacol..

[56] Vale-González C., Gómez-Limia B., Vieytes M.R., Botana L.M. (2007). Effects of the marine phycotoxin palytoxin on neuronal pH in primary cultures of cerebellar granule cells. J. Neurosci. Res..

[57] Barone R., Prisinzano A. (2006). Peculiarità comportamentale del dinoflagellato *Ostreopsis ovata* Fukuyo (Dinophyceae): la strategia del ragno. Naturalista Sicil..

[58] Ciminiello P., Dell’Aversano C., Iacovo E.D., Fattorusso E., Forino M., Tartaglione L., Benedettini G., Onorari M., Serena F., Battocchi C. (2014). First Finding of *Ostreopsis cf. ovata* Toxins in Marine Aerosols. Environ. Sci. Technol..

[59] Aligizaki K., Katikou P., Milandri A., Diogène J. (2011). Occurrence of palytoxin-group toxins in seafood and future strategies to complement the present state of the art. Toxicon.

[60] Pelin M., Zanette C., De Bortoli M., Sosa S., Della Loggia R., Tubaro A., Florio C. (2011). Effects of the marine toxin palytoxin on human skin keratinocytes: Role of ionic imbalance. Toxicology.

[61] Hartmann W.K., Saptharishi N., Yang X.Y., Mitra G., Soman G. (2004). Characterization and analysis of thermal denaturation of antibodies by size exclusion high-performance liquid chromatography with quadruple detection. Anal. Biochem..

[62] Thavarajah R., Mudimbaimannar V.K., Elizabeth J., Rao U.K., Ranganathan K. (2012). Chemical and physical basics of routine formaldehyde fixation. J. Oral Maxillofac. Pathol..

[63] Munday R., Reeve J. (2013). Risk assessment of shellfish toxins. Toxins.

[64] Ciminiello P., Dell’Aversano C., Dello Iacovo E., Fattorusso E., Forino M., Tartaglione L., Rossi R., Soprano V., Capozzo D., Serpe L. (2011). Palytoxin in seafood by liquid chromatography tandem mass spectrometry: Investigation of extraction efficiency and matrix effect. Anal. Bioanal. Chem..

[65] Eurachem Guide: The Fitness for Purpose of Analytical Methods—A Laboratory Guide to Method Validation and Related Topics. https://www.eurachem.org/images/stories/Guides/pdf/MV_guide_2nd_ed_EN.pdf.

