# Supplementary material for: A Novel Sensitive Cell-Based Immunoenzymatic Assay for Palytoxin Quantitation in Mussels
Source: Toxins (Basel). 2018 Aug 14;10(8):329. doi: 10.3390/toxins10080329 (PMC6116170; doi:10.3390/toxins10080329)
Supplement: Supplementary file 1 [file toxins-10-00329-s001.pdf]

Toxins **2018**, *10*, 329; doi: 10.3390/toxins10080329

Supplementary Materials: A Novel Sensitive Cell-Based Immunoenzymatic Assay for Palytoxin Quantitation in Mussels

Marco Pelin, Silvio Sosa, Valentina Brovedani, Laura Fusco, Mark Poli and Aurelia Tubaro

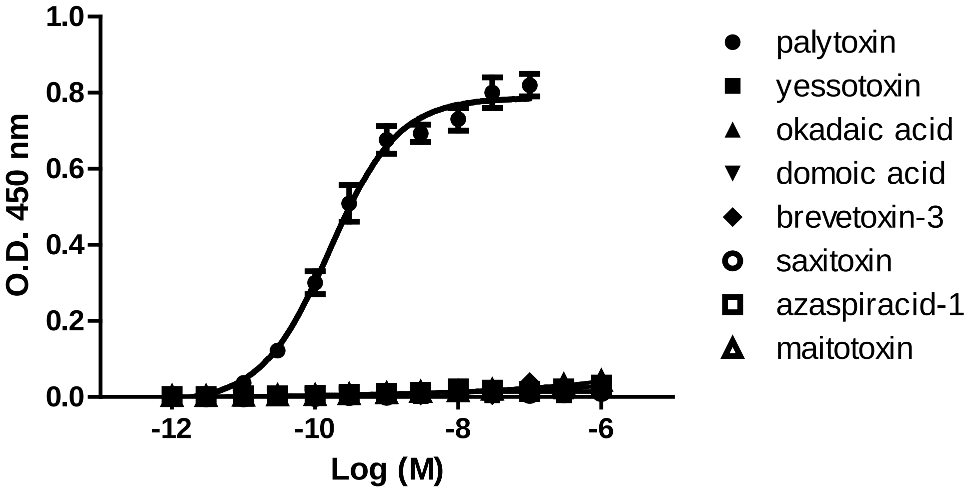

Figure S1. Cross-reactivity of the cell-based ELISA with other marine toxins. Yessotoxin, okadaic acid, domoic acid, brevetoxin-3, saxitoxin, azaspiracid-1, and maitotoxin were analyzed by the cell-based ELISA at concentrations ranging from 1 × 10^{-12} M to 1 × 10^{-6} M. Each point represents mean ± SE of three independent experiments.

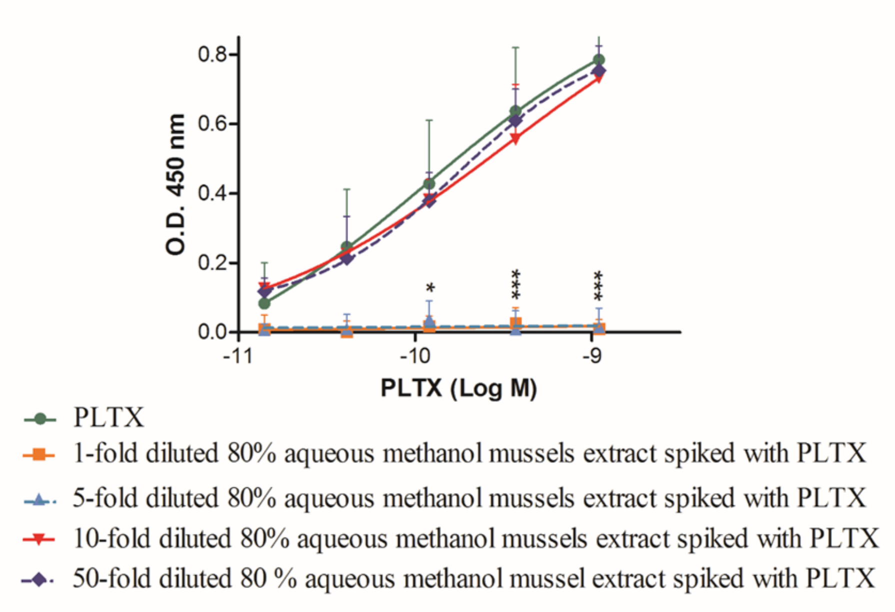

Figure S2. Evaluation of mussel matrix effect on the cell-based ELISA. Comparison between PLTX calibration curve without matrix and 1-fold, 5-fold, 10-fold, and 50-fold diluted 80% aqueous methanol mussel extract spiked with PLTX. Each point represents mean ± SE of three independent experiments. Statistical differences: * $p < 0.05$; *** $p < 0.001$ as compared to PLTX (two-way ANOVA and Bonferroni post test).
